# A drug stabilizable GAL80^ds^ for conditional control of gene expression via GAL4-UAS and CRISPR-Cas9 systems in *Drosophila*

**DOI:** 10.1038/s41598-024-56343-4

**Published:** 2024-03-11

**Authors:** Vaishnavi Kogenaru, Mark Isalan, Manjunatha Kogenaru

**Affiliations:** 1 Ricards Lodge High School, Lake Road, Wimbledon, London, SW19 7HB UK; 2 Department of Life Sciences, Imperial College London, London, SW7 2AZ UK; 3 Imperial College Centre for Synthetic Biology, Imperial College London, London, SW7 2AZ UK; 4 Neuroscience Institute, NYU Langone Medical Center, 435 E 30th St., New York, NY 10016 USA; 5 Present Address: West Windsor-Plainsboro High School South, 346 Clarksville Rd, Princeton Junction, NJ 08550 USA; 6 Present Address: Institute for Systems Genetics, NYU Langone Medical Center, 435 E 30th St., New York, NY 10016 USA

**Keywords:** Protein design, Transgene expression, Gene function, Gene toxicity, Lethality gene, Gene regulation, Genetics

## Abstract

The binary GAL4-UAS expression system has been widely used in *Drosophila* to achieve tissue-specific expression of genes. To further allow for simultaneous spatial and conditional control of gene expression in existing GAL4 expression lines backgrounds, temperature and chemical controllable GAL80 variants have been engineered. Here we add a new drug stabilizable GAL80^ds^ variant, by fusing it to a low-background DHFR-22-DD. We first quantify both single (DD-GAL80) and double (DD-GAL80-DD) architectures and show varied background and activation levels. Next, we demonstrate the utility of GAL80^ds^ *Drosophila* line to regulate a cell death gene ectopically, in a drug-dependent manner, by utilizing an existing tissue-specific GAL4 driver that regulates the expression of a cell death gene under a *UAS*. Finally, we showcase the usefulness of GAL80^ds^ in tight drug-mediated regulation of a target gene, from an endogenous locus, by utilizing an existing tissue-specific GAL4 to drive the expression of a dead Cas9 variant fused to the transcriptional coactivator *nejire,* under a *UAS* and in gRNA lines. Overall, these new GAL80^ds^ lines expand the use of the wide variety of existing tissue-specific GAL4 and gene-specific gRNA lines. This enables conditional control of genes, both ectopically and endogenously, for a broad array of gene expression control applications.

## Introduction

The ability to control the expression of a gene of interest in specific cells or tissues has greatly contributed towards unveiling biological mechanisms in multicellular organisms^1–4^. In the fruit fly *Drosophila melanogaster* model system, this has been achieved by utilizing various exogenous transcription factors and their cognate regulatory operator DNA sequences. Engineered transcription factors with strong activation domains are thus used to drive the expression of a transgene in specific cells or tissues. In these bipartite expression systems, two distinct transgenic lines are created that encode a transcriptional activator under cell-specific or tissue-specific endogenous regulatory elements in a driver line, and a transgene of interest, under the control of exogenous transcription factor specific regulatory elements, in a reporter line. These two genetic components are brought together through simple genetic crossing of driver and reporter parent lines. In the resulting progenies, the transgene is only transcribed in those cells or tissues expressing the transcription factor^5^.

In particular, *Drosophila* bipartite expression systems have utilized transcriptional activator components such as GAL4^6^*,* Tet-On^7^, LexAVP16^8^, and QF^9^*,* under the control of a wide array of cell-specific or tissue-specific driver lines. To utilize these driver lines, corresponding transgenic lines are created by placing a transgene of interest under the control of cognate regulatory elements of the transcriptional activators, such as UAS(GAL4), TetO(TetR), LexAop(LexAVP16) and QUAS(QF). Among these different bipartite expression systems, the GAL4-UAS system has been widely used in *Drosophila,* owing to the availability of large number of GAL4 driver lines. The currently available driver line collection covers a broad array of cell-specific and tissue-specific expression patterns^6,10–12^. Additionally, a vast compendium of UAS-transgene lines has also been generated, hence making the GAL4-UAS system a powerful and versatile tool for the ectopic expression of transgenes.

However, the original GAL4-UAS system only allows for spatial control of transgene expression^6^. Temporal control would further enable the systematic interrogation of the molecular mechanism associated with complex biological systems. To achieve simultaneous spatio-temporal control of transgenes, chemically inducible GAL4 has been engineered^13–15^. These chemically inducible GAL4 versions are developed by tethering the GAL4 DNA binding domain and a transcriptional activation domain, like p65 or VP16, with chemically responsive domains such as progesterone steroid receptor, that responds to the synthetic progesterone analogue inducer mifepristone (RU-486)^13,14^. Alternatively, a Destabilizing Domain (DD) can be used, that responds to the small molecule inducer Trimethoprim^15^. This allows for conditional regulation of the chimeric GAL4 transcription activity, via the presence or absence of the respective small-molecule chemical inducer.

The chemically inducible chimeric GAL4 approach can thus be used to achieve simultaneous spatio-temporal control of a transgene. However, the approach requires generation of new chimeric GAL4 lines, expressed under cell- and tissue-specific enhancers, and is hence not efficient. Instead, to allow for direct use of the existing broad array of currently available cell-specific and tissue-specific expression drivers, and the vast array of UAS-transgene reporter lines, a temperature-sensitive GAL80 (GAL80^ts^) protein has been developed in the TARGET (temporal and regional gene expression targeting) system^16–18^. The transcriptional activation of UAS-transgenes by GAL4 can be inhibited by GAL80^ts^ at 18 °C by binding to the activation domain of GAL4. However, this inhibition can be relieved by raising the temperature to 29 °C, in this way allowing for temporal control of transgene expression via GAL4-UAS expression system. Using a temperature change to achieve spatio-temporal control of a transgene is not ideal for experiments involving behavior, neuropathy and ageing, due to the direct impact of temperature on the overall physiology of living systems^19^.

Subsequently, a range of chemically-inducible GAL80 systems have been developed to overcome the limitation of temperature-induced GAL80^ts^ via the GAL4-UAS expression system^20–22^. In the Tet-off GAL80 system, the GAL80 expression is under the control of a promoter containing tetracycline operator sequences (TetO), to which the ubiquitously expressed tetracycline-responsive transactivator (tTA) binds to regulate the expression level of GAL80. In the absence of inducer, tetracycline, tTA binds to the TetO-regulated promoter. Hence GAL80 expression is switched on, that in turn inhibits GAL4 transcriptional activity, thereby preventing the expression of the UAS-transgene. Upon addition of tetracycline, GAL80 expression is switched off as tTA cannot bind to the TetO promoter. Hence, GAL4 can activate the expression of a UAS-transgene^20^. However, the expression of GAL80 from the tetracycline-inducible promoter is not sufficient to completely inhibit the GAL4 transcriptional activity. Furthermore, the system requires multiple copies of GAL80 insertions, the repression ability of GAL80 declines with age and requires long induction time (> 5 days)^23^.

More recently, the auxin-inducible gene expression system (AGES) has been developed^21^. In this system, GAL80 activity is regulated via drug-dependent degradation. To achieve this, GAL80 is fused to a plant-derived auxin-inducible degron (AID), which, in the presence of phytohormone indole-3-acetic acid (IAA; a natural auxin), binds to an auxin receptor (*Arabidopsis thaliana* TIR1; AtTIR1)^24–27^. This plant-derived AtTIR1 protein can interact with endogenous conserved *Drosophila* proteins Skp1 and Cullin, to form an E3 ligase complex. This complex then ubiquitinates AID, resulting in auxin-dependent degradation of the AID-GAL80 fusion by the ubiquitin–proteasome pathway^28^. GAL4 can thereby activate the expression of UAS-transgene. In the absence of IAA, the GAL80-AID fusion accumulates in the cells. Hence it can inhibit transcriptional activation activity of GAL4 and so the expression of the UAS-transgene is switched off^21^. However, it has been shown that AGES does not achieve a high level of expression of UAS-transgenes, even with very high concentrations of IAA, when compared to the TARGET system^29^.

Here we engineered a new drug-stabilizable GAL80, GAL80^ds^, by fusing the GAL80 to Destabilizing Domains (DDs). The latter conditionally controls the level of abundance of GAL80-DD fusion protein, when combined with the specific stabilizing small-molecule drug, Trimethoprim (TMP). TMP has been shown to have no detectable side effects on the lifespan or behavior of the animal^22^. In particular, we used a DD variant, DHFR-22, that was shown to have the least basal expression in our previous study^15^, when compared to DHFR-07, which was previously fused to GAL80 by Sethi et al.^22^.

Our new GAL80^ds^ system turns UAS-transgene expression off in the presence of the small-molecule TMP. This is different to the Tet-off GAL80 and AGES systems, where UAS-transgene expression is turned on in the presence of their respective small-molecules, to achieve temporal control. Finally, we showcase the usefulness of GAL80^ds^ in making use of the existing wide-array of tissue-specific GAL4 and gene-specific gRNA lines. It is thus possible to achieve conditional control of gene expression, both ectopically and endogenously, for virtually any gene-of-interest.

## Results

### A drug-stabilizable GAL80^ds^ conditionally controls transgene expression in cultured *Drosophila* cells

**Figure 1:**
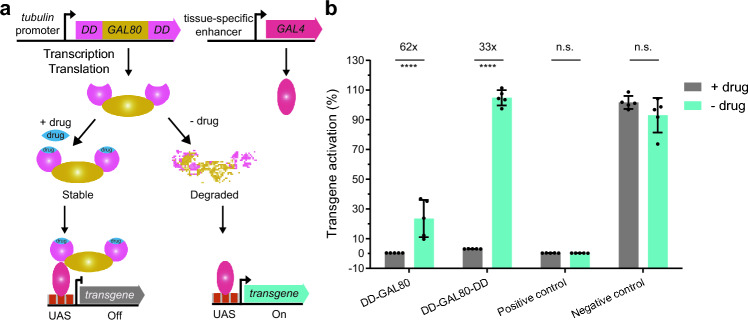
The drug stabilizable GAL80^ds^ conditionally controls transgene expression via the GAL4-UAS bipartite expression system in *Drosophila melanogaster* cultured cells. (**a**) Sketch of the drug stabilizable GAL80^ds^ mode of action. An *αTub84B* tubulin promoter constitutively drives the expression of destabilizing domains (DDs) fused to either N-terminus alone or both N- and C-termini of the GAL80 construct (DD-GAL80 or DD-GAL80-DD; for clarity only, the latter is illustrated). This GAL80^ds^ protein is intrinsically unfolded in the absence of the small molecule drug, Trimethoprim (− drug), and hence can be degraded. The GAL4 transcriptional activator protein, expressed in a specific tissue, can activate the expression of a transgene under the control of an enhancer element Upstream Activating Sequence (*UAS*). However, degradation of GAL80^ds^ fusion protein can be rescued in the presence of Trimethoprim (blue oval, + drug). This stable GAL80^ds^ protein binds to the GAL4 transcriptional activator domain and inhibits its transcriptional activation, resulting in switching off transgene expression. This allows for conditional control of transgene expression in the wide array of tissue-specific GAL4 expression lines that are currently available for *Drosophila*. (**b**) *Drosophila* S2R+ cells with transient expression of these constructs were treated with and without drug, and the *eGFP* transgene activation under the *UAS* enhancer was measured by flow cytometry. The histogram shows the normalized mean eGFP fluorescence transgene activation in the mock treatment with DMSO (− drug) and the presence of 10 µM TMP (+ drug). The positive control consists of GAL80 without DD, while the negative control substituted the GAL80 for Gaussia Luciferase in DMSO and 10 µM TMP conditions. The fold-induction and statistical significance resulting from a t-test are summarized with multiple asterisk marks representing the level of significance (**** = P-value ≤ 0. 0001 and n.s. = P-value > 0.05). Data are represented as mean ± SD from five biological replicates.

We previously applied single and double architectures of the least-background DHFR22-DD to the transcriptional activator GAL4^15^. Thus, we showed small-molecule TMP drug-dependent activation of transgene expression, under a *UAS* promoter in vivo. We sought to extend this to GAL80, the inhibitor of transcriptional activator GAL4. Fusing DHFR22-DD to GAL80 makes its level of abundance be controlled conditionally using the small-molecule drug TMP, at the post-translational level. This enables conditional control of gene expression in *Drosophila* (Fig. 1a). In the absence of the drug, the DHFR22-DD domain is intrinsically unfolded and so the whole DD-GAL80 fusion protein is degraded by the protein quality control machinery. In this condition, any GAL4 transcriptional activator that is expressed from a tissue-specific promoter can activate the expression of a transgene under the *UAS* promoter. However, in the presence of TMP, DHFR22-DD domain folds properly. Hence, the DD-GAL80 whole fusion protein becomes stable and thereby binds to the GAL4 transcriptional activator, inhibiting its transcriptional activity. This results in turning off the transgene expression from *UAS* promoter (Fig. 1a). The main advantage of this approach is that GAL80^ds^ would be compatible with the wide range of tissue-specific GAL4 driver lines that are currently available, thus achieving conditional control of transgene expression.

To assess the function of the single and double architectures of DHFR22 on GAL80, we cloned DHFR22 in frame with GAL80 (DD-GAL80 or DD-GAL80-DD), under a constitutive α-tubulin promoter (see “Methods” and Supplementary Fig. 1). These constructs also co-express a fluorescent protein marker, mCherry, under the same constitutive α-tubulin promoter, making use of the highly efficient self-cleaving 2A peptide sequence from *Thosea asigna* virus (T2A)^30,31^. The plasmid constructs encoding single and double architecture GAL80 DDs were each transiently transfected into *Drosophila* S2R+ cells. The DDs were co-transfected with GAL4 transcriptional activator, co-expressing mTurquoise fluorescent protein marker via T2A, driven by the same constitutive α-tubulin promoter, and a reporter plasmid encoding Green Fluorescent Protein (GFP) under the *UAS* promoter. The resulting fluorescence intensities from mCherry, mTurquoise, and GFP proteins were measured in the absence and presence of TMP drug by flow cytometry.

The DD-GAL80 single architecture construct was able to completely inhibit the transcriptional activity of GAL4; as a result, only 0.4% of the level of expression of transgene GFP was observed in the presence of the drug. This level of expression is comparable with that of a positive control GAL80 without DD (0.3%), suggesting close to complete inhibition of GAL4 transcriptional activity by the single DD-GAL80 architecture. However, transgene activation level could only reach a maximum of 24% of the negative control (created by substituting the GAL80 for Gaussia Luciferase enzyme) (Fig. 1b). This suggests that the single architecture DD-GAL80 is not fully degraded in the absence of TMP. The background level of DD-GAL80 can inhibit the transcriptional activation activity of GAL4, resulting in an overall reduction of the maximum level of transgene expression that is achievable. This observation agrees with our previous study that found ∼3% background expression for DHFR22-DD in absence of TMP drug, in *Drosophila* S2R+ cells^15^.

By contrast, the DD-GAL80-DD double architecture showed a 10% higher expression level of transgene GFP in the presence of the TMP drug, compared to the positive control level, although the level was still low, at 3%. This suggests that double architecture GAL80 construct is not fully able to inhibit transcriptional activation activity of GAL4 in the presence of the drug. By contrast, in the absence of the drug, the double architecture GAL80 construct is fully able to relieve the inhibition of GAL4 transcriptional activity. As a result, transgene activation level can reach the maximum level possible. Overall, the single (DD-GAL80) and double (DD-GAL80-DD) architectures resulted in a 62- and 33-fold difference in the level of transgene expression, in the presence and absence of the drug, respectively (Fig. 1b). Despite differing background and activation levels, both drug stabilizable GAL80^ds^ variants conditionally control transgene expression via the GAL4-UAS bipartite expression system in cultured *Drosophila* cells.

### A drug stabilizable GAL80^ds^ conditionally controls the ectopic expression of a transgene in a whole animal model

An ideal GAL80^ds^ should have 0% transgene expression level in the presence of a drug but should activate transgene expression level back to 100% in the absence of a drug, in the genetic background of the GAL4-UAS bipartite expression system. However, only the single architecture DD-GAL80 shows the desired almost total lack of transgene expression in the presence of a drug, whereas the double architecture GAL80 shows the desired full expression of transgene in the absence of the drug (Fig. 1b). Since no architecture showed ideal GAL80^ds^ construct properties in vitro, we sought to further compare both architectures in vivo. To facilitate the combining of GAL80^ds^ *Drosophila* lines with the existing large number of tissue-specific GAL4 driver lines and UAS-transgene transgenic lines, we placed the expression of GAL80^ds^ constructs under the constitutive *α-tubulin* promoter. We created transgenic fly lines from both single and double architectures of DFHR22-DD GAL80^ds^ constructs, by inserting them into the second and third chromosomes of *Drosophila melanogaster* (see Methods).

**Figure 2:**
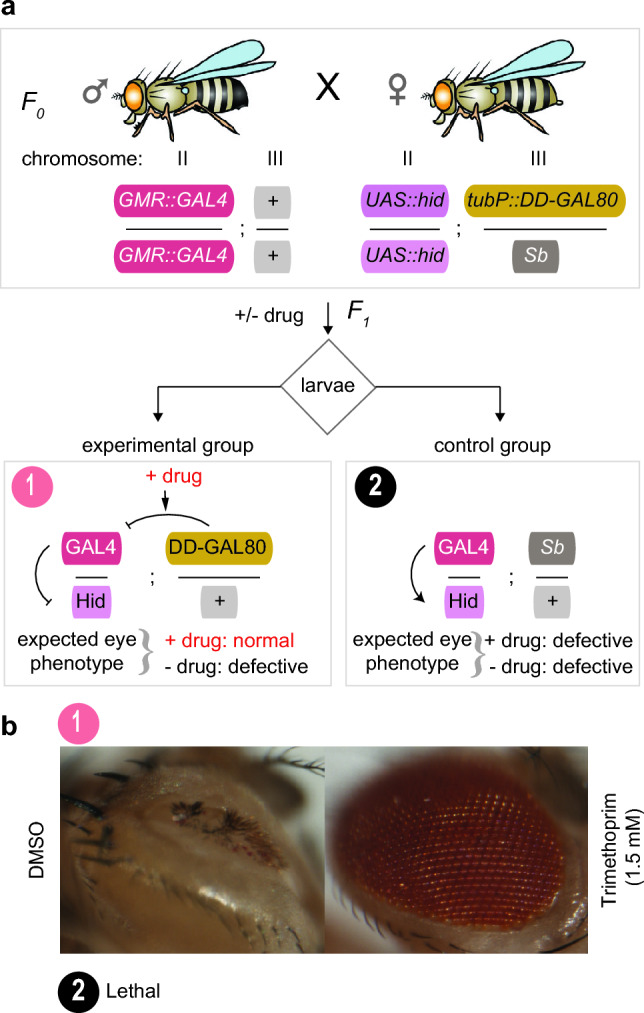
The single DD architecture drug stabilizable Gal80^ds^ conditionally controls ectopic transgene expression via the Gal4-UAS bipartite expression system in vivo*,* in *Drosophila melanogaster*. (**a**) Experimental outline to demonstrate the drug-dependent control of ectopic transgene expression. Schematic representation of the genotypes of transgenic flies. The driver line encodes GAL4 transcriptional activator under the control of eye-specific enhancer, glass multiple reporter (*GAL4*). Meanwhile, the reporter line encodes a pro-apoptotic gene *hid,* expressed under the *UAS*-promoter (*UAS::hid*) on chromosome II. Additionally, GAL80^ds^ contains a DHFR-22-DD fused to the N-terminus that is constitutively driven by an *αTub84B tubulin* promoter and is on chromosome III (*tubP::DD-GAL80*). *F*_*0*_ genetic crosses were set up between the homozygous male *GMR::GAL4*/*GMR::GAL4* driver line with the female reporter line encoding homozygous *UAS::hid/UAS::hid* and heterozygous for *tubP::DD-GAL80/TM3, Sb*. The resulting *F*_*1*_ population of third instar larvae were allowed to feed on standard fly food supplemented with DMSO for mock-treatment (− drug), or 1.5 mM of TMP (+ drug). The box shows the expected genotypes and associated phenotypes of *F*_*1*_ progenies in the absence or presence of drug; conditions are indicated for control and experimental groups of animals. The black arrows indicate the network of interactions between GAL80, GAL4 and *UAS* components. (**b**) Representative images showing conditional structural defects or wild-type eyes. The progenies from the control group (constitutive expression) are not shown as they result in lethality at the pupae stage.

To test single and double architectures of GAL80^ds^ *Drosophila* lines in vivo, we utilized an existing tissue-specific GAL4 driver line under the glass multiple reporter (*GMR*) eye-specific enhancer. This driver line regulates the expression of a pro-apoptotic head involution defective gene, *hid,* under the *UAS* promoter, which executes a cell death pathway that results in structural defects in the eyes of adult flies^32^. To perform this experiment, we first combined the *UAS-hid* reporter genetic component with single (*tubP::DD-GAL80*) or double (*tubP::DD-GAL80-DD*) destabilizing domain components; these were combined into one line by genetic crossing (Fig. 2a). The single and double architecture *GAL80 UAS-hid* transgenic lines were crossed with a homozygous *GMR::GAL4* driver line. The resulting *F*_*1*_ larvae were exposed to mock- and drug-treated conditions. The emerged adult progenies were sorted into control and experimental groups based on their genotypes. The progenies with genotype from the experimental group showed the expected aberrant eye phenotype in adult flies when mock treated with DMSO. This is because GAL80^ds^ degraded in the absence of the TMP drug, and so the GAL4 transcriptional activator could upregulate the *hid* transgene under the *UAS* promoter, resulting in an aberrant eye phenotype in adult flies. Interestingly, the same genotype showed the rescue of defective eye phenotype when treated with 1.5 mM of TMP drug (Fig. 2b). This is because the TMP drug stabilizes the GAL80^ds^, enabling inhibition of GAL4 transcriptional activity: as a result, *hid* transgene expression is turned off and a wild-type eye can develop. Notably, we did not recover any progeny from the control group genotype due to associated lethality of constitutive *hid* expression at the pupae stage, as no adults eclosed from the pupal case. The observed phenotypes for both groups of progenies showed results that were consistent with complete penetrance. We also observed complete lethality at the pupae stage for the double architecture construct. We recovered no adult progenies from both genotypes in either mock or drug-treated conditions.

The non-lethal and lethal phenotypes observed with single and double architectures of GAL80^ds^ respectively, match the data observed in a cell-based quantification (Fig. 1b). The single architecture DD-GAL80 is not completely degraded in absence of drug. This background level of DD-GAL80 can inhibit the transcriptional activation activity of GAL4, resulting in an overall reduction of maximum level of *hid* transgene expression achievable (Fig. 1b). This lower level of expression of the *hid* transgene, in the progenies of experimental group with genotype *GMR::GAL4/UAS::hid; tubP::DD-GAL80/*+*,* may have allowed the progenies to bypass the lethality, but the level of expression is still enough to induce the aberrant eye phenotype in the adult progenies. By contrast, the control group progenies with genotype *GMR::GAL4/UAS::hid; Sb/*+ completely lack the DD-GAL80 inhibitor. Hence the GAL4 was fully able to activate the expression of *hid* transgene to its maximum possible level, and this resulted in complete lethality with no eclosion of adult progenies from the pupal case. On the other hand, in the case of the double architecture, GAL80^ds^ was completely degraded in the absence of TMP drug. This also resulted in the maximum level of *hid* transgene expression, leading to complete lethality. In the presence of the TMP drug, the double architecture showed 10% higher background expression, which might have contributed to complete lethality observed, with no eclosion of adult progenies from the pupal case (Fig. 1b). Taken together, these observations indicate that single architecture DD-GAL80 is more suitable to achieve conditional control of transgene expression ectopically via the GAL4-UAS bipartite expression system.

### A drug stabilizable GAL80^ds^ conditionally controls gene expression at an endogenous locus in conjunction with the CRISPR-dCas9 system

**Figure 3:**
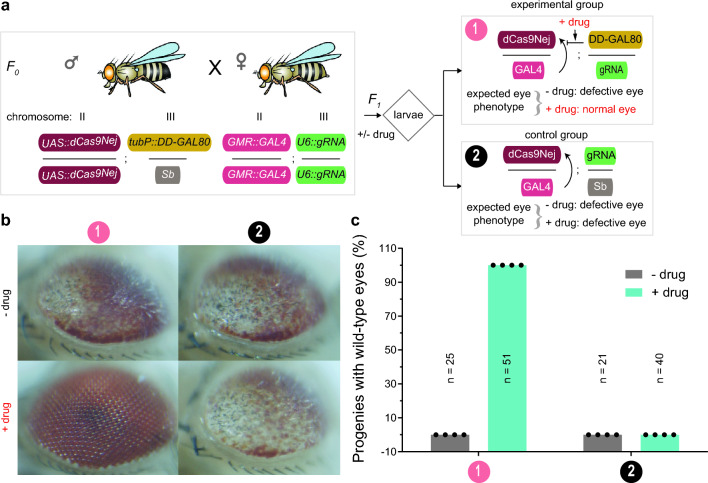
Drug stabilizable GAL80^ds^ conditionally controls gene expression at an endogenous locus via a GAL4-UAS bipartite expression system in conjunction with dCas9Nej and a gRNA targeting the *eve* gene at its endogenous locus, in vivo*,* in *Drosophila melanogaster*. (**a**) Experimental outline to demonstrate the drug-dependent control of endogenous gene expression. Schematic representation of the genotypes of transgenic fly lines used. One transgenic line encoding dead Cas9 fused to *Nejire* core, under the *UAS*-promoter on chromosome II; GAL80^ds^, DD fused to the N-terminus that is constitutively driven by an *αTub84B* tubulin promoter on chromosome III (*tubP::DD-GAL80*). The second transgenic line encoding GAL4 transcriptional activator under the control of eye-specific enhancer, glass multiple reporter (*GMR::GAL4*) on chromosome II; a guide RNA (gRNA) targeting *eve* gene promoter under the *U6::3* promoter (*U6::gRNA-eve*) on chromosome III. *F*_*0*_ genetic crosses were set up between the two transgenic lines. The resulting *F*_*1*_ population of third instar larvae were allowed to feed on standard fly food supplemented with DMSO for mock-treatment (− drug) or 1.5 mM of TMP (+ drug). Adult flies emerging from these treatments were sorted into two groups based on the genotypes and visually scored for eye defects under the bright-field microscope. The expected genotypes and associated phenotypes of *F*_*1*_ progenies, in the absence or presence of drug conditions, are indicated for two groups of animals. An arrow in the genotype notations indicates the cascade of activation, or no activation, of downstream genes or proteins. (**b**) Representative images showing structural defects and wild-type eye phenotypes in the adult flies are displayed. A negative control population lacking the *tubP::DD-GAL80* transgene was derived from the Stubble bristle phenotype resulting from a dominant *Sb* marker from the heterozygous *tubP::DD-GAL80/TM3, Sb* line. (**c**) Quantification of the rescued structural defects observed in the adult *Drosophila* eyes. The total numbers of individual flies (n) examined in each data set are indicated. Data presented from four biological replicates which gave consistently the same proportions of eye defects or wild-type eyes.

The CRISPR-Cas9 system has emerged as a powerful technology for tissue-specific mutagenesis or control of gene expression at endogenous loci in *Drosophila*^33–43^. For this purpose, tissue-specific GAL4 lines have been used to drive Cas9 under *UAS*, in specific tissues or cell types, and combine this with transgenic expression of a guide-RNA (gRNA) targeting a gene of interest^43^. We therefore next combined the drug stabilizable GAL80^ds^ with the CRISPR-Cas9 and UAS-GAL4 system to conditionally control target gene expression at endogenous loci. Towards this, we used a 'dead' Cas9 (dCas9) variant, under a *UAS* promoter, fused to *Nejire* core (dCas9Nej), as a transcriptional activator (CRISPRa)^44^. The *Nejire* core is a *Drosophila* ortholog of histone acetyltransferase P300 from *Homo sapiens*. The dCas9Nej has shown to have stronger activation of target genes compared to dCas9-VPR, a tripartite activator consisting of VP64-p65-rta domains, which was previously shown to be most optimal activator in *Drosophila*^45,46^. The dCas9Nej driven by the eye-specific GAL4 driver (*GMR::GAL4*), combined with gRNA targeting *eve* promoter (gRNA-eve) shown to have 863-fold upregulation of *eve* gene^44^. The overexpression of *eve* gene in the eye of *Drosophila* using this system is known to result in an aberrant eye phenotype^44^. Moreover, we added the drug stabilizable GAL80^ds^ system to this genetic background and tested for drug-dependent rescue of the observed aberrant eye phenotype (Fig. 3).

To perform this experiment, we first combined the *UAS-dCas9Nej* and *tubP::DD-GAL80* genetic components into one line by genetic crossing (Fig. 3a). Similarly, the *GMR::GAL4* driver and *U6::gRNA-eve* targeting *eve* promoter components were brought into a second line by genetic crossing, utilizing the various transformation markers. These two transgenic lines were then crossed. The resulting *F*_*1*_ larvae were exposed to mock- and drug-treated conditions. The adult progenies that eclosed from the pupal case were sorted into control and experimental groups based on their genotypes (Fig. 3a).

The progenies with genotype from the experimental group showed the expected aberrant eye phenotype when mock treated with DMSO. This is because in the absence of the TMP drug, the GAL80^ds^ protein is degraded. Hence, the GAL4 transcriptional activator can activate the expression of *dCas9Nej*, which upregulates *eve* gene expression, in the presence of gRNA, leading to an aberrant eye phenotype. However, in the presence of TMP drug, GAL80^ds^ can inhibit the GAL4 transcriptional activity leading to turning off the *dCas9Nej* expression and resulting in a normal eye phenotype (Fig. 3b). The progenies with genotypes from the control group showed the expected aberrant eye phenotype in both the absence and presence of TMP drug, due to a complete lack of the GAL80^ds^ inhibitor component (Fig. 3b). The observed phenotypes from the control and experimental groups showed complete penetrance in the population (Fig. 3c). Overall, this experiment suggests that drug stabilizable GAL80^ds^ can conditionally control target gene expression at endogenous loci via the GAL4-UAS bipartite expression system, in conjunction with dCas9Nej and gRNA targeting a gene of interest.

## Discussion

In this study, we characterized the single and double architectures of DHFR-22 DD fusion on GAL80 to achieve drug-mediated conditional control of target gene expression via GAL4-UAS and CRISPR-Cas9 systems in *Drosophila melanogaster*. We first compared the two constructs directly by making use of a previously developed quantification method^15^. We found that background expression of the single architecture DD-GAL80 construct, in the absence of TMP drug, is essential to completely inhibit the transcriptional activity of GAL4; this resulted in no background expression of a target gene under the *UAS* promoter (Fig. 1b). With TMP drug, this single architecture GAL80^ds^ showed only partial activation of target gene expression. This also turned out to be an advantage for studying lethal genes at whole organism level because this led to the survival of the organism, while displaying the associated phenotype in the eye, thus allowing the study of the phenotype in detail (Figs. 2b & 3b). The full expression of such a lethal gene leads to complete lethality, preventing the opportunity to study the causes and consequences of temporal expression in a specific tissue.

The drug stabilizable GAL80^ds^ lines that we created in this study ubiquitously express GAL80^ds^ from *α-tubulin* promoter. Hence, these lines are compatible in combination with a wide variety of tissue-specific GAL4 lines, to achieve conditional control of target gene expression across diverse tissue or cell types in *Drosophila*. For comparison, the GAL80^ds^ line created by Sethi et al., is expressed from a pan-neuronal promoter, *n-synaptobrevin* (nsyb), restricting its use only to the fly brain to achieve conditional control of target gene expression^22^. Moreover, Sethi et al. used the DHFR-07 variant to develop drug stabilizable GAL80^ds^ line, which is known to have higher background than DHFR-22 DD, as shown in our previous study^15^. However, Sethi et al., have thoroughly characterized the kinetics of the TMP drug and its impact on a DD fused to GAL80 or GFP proteins in the *Drosophila* adult fly brain. They demonstrated that it would take approximately 24 h for TMP to reach the brain and stabilize the DD fusion proteins after feeding with food containing TMP. Also, the GAL80-DD fusion protein began to destabilize at approximately 24 h post removal of flies from TMP-containing food to regular food, but complete destabilization was achieved at ~ 72 h. Since our systems also use a TMP-controlled DD fused to GAL80, the TMP kinetics and their impact on GAL80 would be expected to be similar to the findings of Sethi et al. We therefore anticipate that it would take ~ 24 h in our DD-system to conditionally activate or inactivate a gene of interest in the brain.

We demonstrated the use of GAL80^ds^ *Drosophila melanogaster* transgenic lines by combining with a tissue-specific GAL4 driver line to restrict Cas9 expression to a desired group of cells or tissue, to achieve conditional or spatiotemporal control of a target gene at its endogenous locus. CRISPR-Cas9-based transcriptional activation, repression or mutagenesis has recently emerged as a powerful and scalable method for control of gene expression or mutagenesis analysis in *Drosophila*^33–43^. The GAL80^ds^ *Drosophila* transgenic lines that we created can therefore be exploited to achieve tissue-specific or germline mutagenesis of target genes in addition to controlling their expression in a temporal fashion. Target gene expression control or mutagenesis at endogenous loci has vastly contributed to the understanding of various biological mechanisms. The temporal studies would further enhance the understanding of biological processes.

Our GAL80^ds^ system turns target gene expression off in the presence of the small-molecule TMP drug. This contrasts with other currently existing drug-mediated GAL80 systems, like the Tet-off GAL80 and AGES systems, where target gene expression is turned on in the presence of their respective small-molecule to achieve temporal control. Hence, our GAL80^ds^ system provides a complementary approach to achieve conditional control of target gene expression. Finally, like all other chemically mediated GAL80 control systems, the tissue-specific GAL4 line must encode the wild-type GAL4 activation domain to be compatible with GAL80^ds^ lines that are developed in this work. In conclusion, the drug stabilizable GAL80^ds^ lines that we developed in this study expand the use of the many existing tissue-specific GAL4, gene-specific gRNA and RNAi lines, to achieve conditional control of expression level, or mutagenesis, or knockdown of target genes, for a wide variety of applications.

## Methods

### DNA constructs

The *DHFR22-DD* (Addgene plasmid #47076) DNA sequence was fused either to 5ʹ or 3ʹ of the *GAL80* (Addgene plasmid #62953) gene sequence (Supplementary Fig. 1) by overlap PCR extension and was further fused to the self-cleaving peptide sequence *T2A*^30,31^. Similarly, the positive control *GAL80* without *DHFR22-DD* and a negative control encoding *Gaussia Luciferase* (*gLuc*) were PCR amplified along with the *T2A* sequence. These full-length PCR products were cloned into an entry vector, pB1tubP-TGVmChe1B made in house, by utilizing BamHI and XbaI restriction sites, which encodes upstream constitutive *αTub84B* tubulin promoter (tubP) and downstream mCherry reporter with respect to the cloned sequences. The expression cassettes in these plasmids were flanked by short 40 base pairs (bps) *attB* recombinase sites^47^, allowing site specific integration into the genome via ΦC31 recombinase mediated cassette exchange.

The *GAL4* (Addgene plasmid #62956) DNA sequence was PCR amplified and was further fused to *T2A, mTurquoise,* and simian virus 40 polyadenylation signal sequences by overlap PCR extension. The resulting full-length PCR product was cloned into an entry vector, pB1tubP-GAL4dT2AmChe1B made in house, by utilizing BamHI and NdeI restriction sites. A reporter construct encoding *eGFP* under the upstream activating sequence (*UAS*) promoter was previously described^48^. All the DNA constructs were verified by Sanger sequencing.

### Cell culture and transfection

*Drosophila* S2R+ cells were obtained from the *Drosophila* Genome Resource Center (DGRC). Cells were cultured in *Drosophila* Schneider’s medium (Gibco), supplemented with 10% of fetal bovine serum (FBS), 1% of penicillin/streptomycin, at room temperature, in a humidified chamber.

Transfections were performed using Effectene reagent (Qiagen), following the manufacturer’s instructions, using 0.1 µg of DNA with a DNA:Enhancer ratio of 1:8 and DNA:Effectene ratio of 1:15. 50 µL of the transfection complexes were added to each well in a 96-well plate seeded with 1.5 × 10^5^ cells in 150 µL of medium. All transfections were performed in quintuplicates.

### Drug treatment and flow cytometry

Cell cultures were either mock treated with 2 µL of Dimethyl sulfoxide (DMSO) or with 2 µL of 100× concentrated Trimethoprim (TMP) drug solution in DMSO, to achieve the final concentration of 0.1 mM. The culture plate was incubated for 72 h before harvesting for measuring the fluorescence by flow cytometry. The fluorescence measurements were performed on a BD LSRFortessa cell analyzer flow cytometer. The mTurquoise, eGFP and mCherry fluorescence were measured using a 405–470/20, 488–530/30 and 561–610/20 nm excitation lasers and emission filters, respectively. 30,000 living cells, positive for mCherry fluorescence, were measured from each sample. The compensation for the possible crosstalk of fluorescence measurements was corrected using single fluorophore controls in FlowJo 10.0.7r2 software (BD Life Sciences). From the single-cell compensated fluorescence intensities, we further computed the mean fluorescence intensity per cell, representing the population average for each fluorescent marker separately using the FlowJo 10.0.7r2 software (BD Life Sciences). The mean eGFP fluorescence values were further normalized to mCherry fluorescence intensities after substracting for auto-fluorescence derived from mock-transfected cells. The resulting ratiometric scores were further converted to %, based on the average ratiometric score of the negative control samples^15^.

### Generation of GAL80^ds^ transgenic fly lines

The target sites for RMCE exchanges were y1 w*; P{attP.w + .attP}JB37B (Bloomington Drosophila Stock Center, BDSC#27387) and y1 w*; P{attP.w + .attP}JB89B (BDSC#25091) to generate second and third chromosome stable insertions, respectively^49^. These loci contain the mini-white gene, flanked by inverted *attP* sites (a gift from J Bateman and T Wu). Insertion was carried out using ΦC31 recombinase mediated cassette exchange at BestGene Inc. (Chino Hills, CA, USA). The resulting DD-GAL80 lines are deposited at BDSC and the genotypes are as follows: y[1] w[*]; P{tubP-GAL80.1xDHFR22-T2A-mCh}JB37B/CyO (BDSC#600209), and w[*]; sna[Sco]/CyO; P{tubP-GAL80.1xDHFR22-T2A-mCh}JB89B/TM3, Sb[1] (BDSC#600212). In addition to the transgenic fly lines created in this study, we used four previously created lines: *GMR::GAL4* BDSC# 8605 (a gift from C Desplan), *UAS::hid* (created in house), *UAS::dCas9Nej* (a gift from N Windbichler)^44^ and *U6::gRNA-eve* (a gift from N Windbichler)^44^ to bring GAL80^ds^ into these genetic backgrounds. The resulting GAL80^ds^ lines are also deposited at BDSC and the genotypes are as follows: w[*]; P{y[+ t*] w[+ mC] = UAS-hid.K}VIE-260B; P{tubP-GAL80.1xDHFR22-T2A-mCh}JB89B/TM3, Sb[1] (BDSC# 600210) and w[*]; P{y[+ t7.7] w[+ mC] = UAS-3xFLAG-dCas9::nej(core)}attP40; P{tubP-GAL80.1xDHFR22-T2A-mCh}JB89B/TM3, Sb[1] (BDSC# 600211).

### Protein induction in flies

Flies were reared at room temperature and raised on standard food. Standard fly food (polenta 2%, agar 0.8%, brewer's yeast 10%, fructose 8%, 0.5% of 15% Nipagin in ethanol, 0.75% Propionic acid) was mixed with 100× concentrated TMP, to achieve the final concentration of 1.5 mM, after liquefying the food in the microwave. For experiments involving rescuing of eye structural defects, early third instar larvae (from heterozygote female or male *UAS::hid/UAS::hid;tubP::DD-GAL80/TM3, Sb* with male or female homozygote *GMR::GAL4/GMR::GAL4* line or *UAS::dCa9Nej/UAS::dCa9Nej;tubP::DD-GAL80/TM3, Sb* with *GMR::GAL4/GMR::GAL4;U6::gRNA-eve/U6::gRNA-eve* crosses) were inoculated into food vials with DMSO or 1.5 mM TMP. Larvae were incubated in these vials until the eclosion of adult flies. Emerged adult flies were scored for eye structural defects and representative flies were imaged.

### Supplementary Information

Supplementary Figure 1.

## Data Availability

The *Drosophila melanogaster* transgenic lines are deposited at Bloomington Drosophila Stock Center (BDSC# 600209, BDSC# 600210, BDSC# 600211, and BDSC# 600212).
