# Supplementary material for: A drug stabilizable GAL80ds for conditional control of gene expression via GAL4-UAS and CRISPR-Cas9 systems in Drosophila
Source: Sci Rep. 2024 Mar 11;14:5893. doi: 10.1038/s41598-024-56343-4 (PMC10928143; doi:10.1038/s41598-024-56343-4)
Supplement: Supplementary file 1 — Supplementary Figure 1. [file 41598_2024_56343_MOESM1_ESM.pdf]

Supplementary information

A drug stabilizable GAL80^{ds} for conditional control of gene expression via GAL4-UAS and CRISPR-dCas9 systems for *Drosophila*

Vaishnavi Kogenaru,[1,2] Mark Isalan[3,4] and Manjunatha Kogenaru[3,4,5,6]*

[1]Ricards Lodge High School, Lake Road, Wimbledon, London, SW19 7HB, United Kingdom.
[2]West Windsor-Plainsboro High School South, 346 Clarksville Rd, Princeton Junction, NJ 08550, USA.
[3]Department of Life Sciences, Imperial College London, London, SW7 2AZ, United Kingdom.
[4]Imperial College Centre for Synthetic Biology, Imperial College London, London, SW7 2AZ, United Kingdom.
[5]Neuroscience Institute, NYU Langone Medical Center, 435 E 30th St., New York, NY 10016, USA.
[6]Present address: Institute for Systems Genetics, NYU Langone Medical Center, 435 E 30th St., New York, NY 10016, USA.

*Correspondence: Manjunatha.Kogenaru@nyulangone.org

attB40 recombinase site for ΦC31 recombinase mediated cassette exchange
αTub84B tubulin promoter
SV40 nuclear localization signal (NLS)
ecDHFR22 destabilizing domain (DD)
GAL80 inhibitor of Gal4 transcriptional activator
T2A 2A self-cleaving peptide
mCherry fluorescent marker
SV40 late polyadenylation signal

a - single architecture

CGGGTGCCAGGGCGTGCCCTTGGGCTCCCCGGGCGCGTACGCTAGCGCTACTC*AGATCTTAAGAAACCATTATTA*
TCATGACATTAACCTATAAAAATAGGCGTATCACGAGGCCCTTTCGTCTTCAAGGAATTCGATATCAAGCTTGCA
CAGGTCCTGTTCGATAACGTCGTACTCGGGAATCACCTTTAACTTGTCCGCTATTGGGTATTTGTCCTTAAACAC
ATTCGTAATCTCTCCCACAATCATTGCCCTGGCCTCTGCAATGTAAAAAGTATTTATAAAATATCGCTGGCTCTA
AAGGAAACATATTAATCAAAGTATTGAGGCTTTATCAATCTAACATTTACAAGTAGTCAGACCACAATTTTTAGA
CTGCACTTCTGCGTAAGTAAATGAAATGAAATGGTGACCTACTCTTGGGTAGCCGTACTGTTATATGCTGACGAG
CCTTCACCTGGGCCTGCTTTTTCTCCTGTTTCGTCTGATGCGTCGGATGCGGCTGGACTGGCTGATTGTTGGGAT
TGGGTTTTGGATGCTGCTCCGAAAGGTGGGCCACAGCTTTTGCGATGGTGGTGCCCACTATGCGGTTGCTGCAGG
AACTACAGCTGAATGTTCGCTTTATTGTCTGCAGCTCCTGGGGCTGGACGAAGGCCACGCCCTCACCCGGTGCGG
CCATCTCCTGCGGCAAAACGTATGTGGGATTCCTGTCCAAGTAGCTCTCGATGCCCACCATAATGCGCTCCAGCA
GGTTGGCCAGGTGCGGGTCTAGCTGCGGCGGATTCGTCGTCTGGTTCACTGAGTTTAGGGTCTGCAGAAAGAAGT
GTGCCTCCATGGTAAGCGGATTCATCTGCACGATCAGCGGGCTTTAGTCTAAAAATTCGAATGATACAAACTCGT
ACTCACGTAAAGCAGTTTGTTTGGCATCATCTCGGCCATCGTCTTATTCTTCTTCGTGGCCTTTGTTACCGGAGC
GGCTATGTTGGGCGCGGTGGAGCCCGGAAGCTGAAAGGTTAATCGAATGCGTTAACTTTTCTGCAACTCGAAAGT
TTGCCGCCTTTGTTCGACTGCCAATAACTGTTGATTCGAAAATTCGAATCGAAGCGGTTGAATTTCGTAGGGTGG
CCAACTACACCAAAGTTCGCCGGCGGTGTATGCATGTGAGTGTGAGTGTGACAGTGGGTGTACGTGTGCTTTAAG
CGACACAATCGCACCGCGATGTACATATTTTTGTAGTAATGTGTGTGCATTTTTCGTCCGGATCACGGCTCCATA
AAATAAATCATATCACAGGGCTCAGGGATTTGTGAGTAGAAGTTCCCGCCACTTAAACGTCACTTGTGAAATCGC
CATCGACGGTCAACTTTTACTCACACTGGTCTTTCGCGGACGGACCGTCTCAAAGTACTGCCTTTCTGCGTTGGA
AAACATCGCCTTTTTCGTCCAAAAGGAGTCCCCAGGTTCGATCCGCATGGCGTTGTGCGTGCGTGCCTTTCTTTT
CAAATGATTACGGCTATTAACTTGGGGGCGTTAAGTTGGAAACACGTAAATTGCAGACTGCGATTAGAGTGACCA
TGAGTAGGAGTTCAAAATCTCCTGACATCATTTTCTTAAAACCTGCTTTGTTTTTTACATTTCTATTTAATATAA
CTCCTATTTGAATAAAAAAACAAAACAAGTTTAGATGTTAAGATATTAACTACATCCTTTGCTCCAAAGGGAGAG

GGGAAGTTATGGAGTTAATTAATTTGCTGTTGGAAATCAATATGGAGTCAGAAATATAATGATTTACTAAACCTT
ATTGAATCGGTAACGATGCGAATTTATATTAAAATAGCTTTTATGAAACATTCAACAAAAATATTATTAATGTTG
GCCCACTTTAGCAACCGGTTAGGTCTACCGGTTGGGCAAGCAAAGATTCACGCCCTGGTTCGAGTCCCAACTAGT
CCTGCAAAATACCGCAGCAAGTTTTAGAGAGACCAAGTGCCATTACCTCTCCCACTTCAGTTATCGGTTATGCGG
CGTTTAAGTCGACAGCTTGCCGTCTCTAGCTCCGGTGCCTATATAAAGCAGCCCGCTTTCCACATTTCATATTCG
TTTTACGTTTGTCAAGCCTCATAGCCGGCAGTTCGAACGTATACGCTCTCTGAGTCAGACCTCGAAATCGTAGCT
CTACACAATTCTGTGAATTTTCCTTGTCGCGTGTGAAACACTTCCAATAAAAACTCCTGCAGGTGAGTACTTTAA
AAAAAAATCTAGTGAAATAATGCTGAAAAGAAATTTGTGTGGGCAAAATTCAATGGGCAAAAACGCGATGCGGCT
TTTTCTCAAAATGGCGGCCGGCCTGCGTTTTTTCCTCAAAAGTGATGACGTCATGCCTGTTTTTTTTTTTTTTGTT
CGCAATGAGGAATGGCTCTTAAAATCTACTAGATAAAAAAAATATTCATTATTTCTATGCTGCTGGAACGCTTCA
TTAATCTTAAAAATTCTAAATTCGGTTACCATGATACTTCGACGCATAACTGTAGATTTTGGATAGAATTAAAGA
GAAAATGGCGAGAGAGTAAAATTCCGGCGTCGGCAAAGTAGAGCAAAAAAATCAGTATACCATTTAGCTACCTCT
CTCACTCGCACGCAGTGCCGGCTCAAGTTGGGCGCGGCTCTGCAATTATCGATTTTCTTGGGGTGTGTAACTAAT
*CATCCGTTTTCCCTTCCTCCTCATCCACAGCGTGAA*GGTACCGCGGGCCCGGGATCCACCGGTAATCAAC**ATGCC**
ACCCAAGAAGAAGCGAAAAGTAATGATCAGTCTGATTGCGGCGTTAGCGGTAGATCACGTTATCGGCATGGAAAA
CGCCATGCCGTGGAGCCTGCCTGCCGATCTCGCCTGGTTTAAACGCAACACCTTAAATAAACCCGTGATTATGGG
CCGCCATACCTGGGAATCAATCGGTCGTCCGTTGCCAGGACGCAAAAACATTATCCTCAGCAGTCAACCGAGTAC
GGACGATCGCGTAACGTGGGTGAAGTCGGCGGATGAAGCCATCGCGGCGTGTGGTGACGTACCAGAAATCATGGT
GATTGGCGGCGGTCGCGTTTATGAGCAGTTCTTGCCAAAAGCGCAAAAACTGTATCTGACGCATATCGACGCAGA
AGTGGGAGGCGACACCCATTTCCCGGATTACGAGCCGGATGACTGGGGATCGGTATTCAGCGAATTCCACGATGC
TGATGCGCAGAACTCTCACAGCTATTGCTTTGTGATTCTGGGGCGGCGA**ATGGATTACAACAAAAGGAGTAGTGT**
GAGTACGGTGCCGAATGCTGCTCCCATTCGCGTGGGCTTCGTGGGATTGAACGCGGCTAAGGGTTGGGCCATTAA
AACGCATTATCCAGCCATACTGCAGCTGAGCTCCCAGTTCCAAATAACAGCGCTTTATTCCCCCAAGATCGAGAC
GTCCATTGCGACAATTCAGAGGCTGAAGTTGTCCAACGCCACAGCTTTCCCAACACTCGAGAGCTTCGCCTCGAG
CTCCACGATCGATATGATTGTGATCGCAATCCAGGTGGCTTCCCACTACGAGGTGGTAATGCCCCTGCTCGAGTT
TTCGAAGAACAATCCTAATCTGAAGTATTTGTTTGTGGAATGGGCACTGGCCTGCTCGCTCGATCAGGCGGAGTC
GATCTATAAGGCCGCTGCAGAGCGGGGCGTGCAAACAATAATCAGTCTGCAGGGACGAAAGAGCCCGTACATTTT
GCGGGCAAAAGAGCTTATCTCCCAGGGCTACATCGGCGATATAAATAGCATCGAGATTGCAGGTAACGGAGGTTG
GTACGGTTACGAGCGGCCGGTGAAAAGCCCGAAATACATTTACGAGATCGGAAACGGAGTTGATCTGGTGACCAC
CACGTTCGGTCACACGATAGATATATTGCAGTACATGACCAGTTCGTACTTCAGCCGTATCAATGCCATGGTGTT
TAACAACATTCCAGAACAGGAGCTCATTGATGAGAGGGGCAATCGCCTGGGCCAGCGGGTCCCAAAGACTGTCCC
AGATCATCTCTTGTTCCAGGGCACGCTGCTCAACGGAAATGTTCCTGTGTCCTGTTCCTTTAAGGGAGGCAAGCC
TACGAAAAAGTTCACCAAGAATCTGGTCATAGATATCCATGGTACAAAGGGTGATCTGAAGCTGGAGGGTGACGC
TGGATTTGCGGAAATCTCGAATCTCGTTCTGTATTATTCGGGCACCCGCGCCAATGATTTTCCCCTGGCCAATGG
TCAGCAGGCACCCTTGGACCCCGGCTACGACGCAGGCAAGGAGATTATGGAGGTGTATCACCTCCGAAACTACAA
CGCGATCGTTGGTAATATACACCGGCTCTACCAGAGTATCAGCGATTTCCACTTCAACACCAAGAAAATCCCCGA
GCTTCCATCGCAGTTCGTAATGCAAGGCTTCGATTTCGAAGGCTTTCCCACCCTGATGGATGCCTTGATCTTGCA
TCGCCTGATTGAAAGTGTCTACAAGTCGAATATGATGGGCAGTACCCTGAATGTTTCCAACATCTCCCACTACTC
GCTTGGCGGCCGCGAAGGACGCGGCAGCCTACTGACTTGCGGAGATGTCGAAGAGAACCCTGGCCCTGGTTCCGA
TATCTCTAGAGCCACC*GTGTCCAAGGGCGAAGAGGACAACATGGCCATCATCAAGGAGTTCATGCGGTTCAAGGT*
GCACATGGAAGGCAGCGTGAACGGCCACGAGTTCGAGATCGAGGGCGAGGGCGAAGGCAGGCCCTACGAGGGCAC
CCAGACCGCCAAGCTGAAGGTGACCAAGGGCGGACCTCTGCCCTTCGCCTGGGACATCCTGAGCCCCCAGTTCAT
GTACGGCAGCAAGGCCTACGTGAAGCACCCCGCCGACATCCCCGACTACCTGAAGCTGTCCTTCCCCGAGGGATT
CAAATGGGAGCGGGTGATGAACTTCGAGGACGGCGGCGTGGTGACCGTGACCCAGGACAGCAGCCTGCAGGACGG
CGAGTTCATCTACAAGGTGAAACTGCGGGGCACCAACTTCCCCAGCGACGGCCCCGTGATGCAGAAAAAGACCAT
GGGCTGGGAGGCCAGCAGCGAGCGGATGTACCCCGAGGATGGCGCCCTGAAGGGCGAGATCAAGCAGCGGCTGAA
GCTGAAGGATGGCGGCCACTACGACGCCGAGGTGAAAACCACCTACAAGGCCAAGAAACCCGTGCAGCTGCCTGG
CGCCTACAACGTGAACATCAAGCTGGACATCACCAGCCACAACGAGGACTACACCATCGTGGAGCAGTACGAGCG
*GGCCGAGGGCAGACACAGCACCGGCGGCATGGACGAGCTGTATAAATG*AAGCTTAGACATGATAAGATACATTGA
TGAGTTTGGACAAACCACAACTAGAATGCAGTGAAAAAAATGCTTTATTTGTGAAATTTGTGATGCTATTGCTTT
ATTTGTAACCATTATAAGCTGCAATAAACAAGTTAACAACAACAATTGCATTCATTTTATGTTTCAGGTTCAGGG
GGAGGTGTGGGAGGTTTTTTAAAGCAAGTAAAACCTCTACAAATGTGGTATGGCTGATTATGATCACATATG**GTA**
CGCGCCCGGGGAGCCCAAGGGCACGCCCTGGCACCCG

b - double architecture

CGGGTGCCAGGGCGTGCCCTTGGGCTCCCCGGGCGCGTACGCTAGCGCTACTC*AGATCTTAAGAAACCATTATTA*
TCATGACATTAACCTATAAAAATAGGCGTATCACGAGGCCCTTTCGTCTTCAAGGAATTCGATATCAAGCTTGCA
CAGGTCCTGTTCGATAACGTCGTACTCGGGAATCACCTTTAACTTGTCCGCTATTGGGTATTTGTCCTTAAACAC
ATTCGTAATCTCTCCCACAATCATTGCCCTGGCCTCTGCAATGTAAAAAGTATTTATAAAATATCGCTGGCTCTA
AAGGAAACATATTAATCAAAGTATTGAGGCTTTATCAATCTAACATTTACAAGTAGTCAGACCACAATTTTTAGA
CTGCACTTCTGCGTAAGTAAATGAAATGAAATGGTGACCTACTCTTGGGTAGCCGTACTGTTATATGCTGACGAG
CCTTCACCTGGGCCTGCTTTTTCTCCTGTTTCGTCTGATGCGTCGGATGCGGCTGGACTGGCTGATTGTTGGGAT
TGGGTTTTGGATGCTGCTCCGAAAGGTGGGCCACAGCTTTTGCGATGGTGGTGCCCACTATGCGGTTGCTGCAGG
AACTACAGCTGAATGTTCGCTTTATTGTCTGCAGCTCCTGGGGCTGGACGAAGGCCACGCCCTCACCCGGTGCGG
CCATCTCCTGCGGCAAAACGTATGTGGGATTCCTGTCCAAGTAGCTCTCGATGCCCACCATAATGCGCTCCAGCA
GGTTGGCCAGGTGCGGGTCTAGCTGCGGCGGATTCGTCGTCTGGTTCACTGAGTTTAGGGTCTGCAGAAAGAAGT
GTGCCTCCATGGTAAGCGGATTCATCTGCACGATCAGCGGGCTTTAGTCTAAAAATTCGAATGATACAAACTCGT
ACTCACGTAAAGCAGTTTGTTTGGCATCATCTCGGCCATCGTCTTATTCTTCTTCGTGGCCTTTGTTACCGGAGC
GGCTATGTTGGGCGCGGTGGAGCCCGGAAGCTGAAAGGTTAATCGAATGCGTTAACTTTTCTGCAACTCGAAAGT
TTGCCGCCTTTGTTCGACTGCCAATAACTGTTGATTCGAAAATTCGAATCGAAGCGGTTGAATTTCGTAGGGTGG
CCAACTACACCAAAGTTCGCCGGCGGTGTATGCATGTGAGTGTGAGTGTGACAGTGGGTGTACGTGTGCTTTAAG
CGACACAATCGCACCGCGATGTACATATTTTTGTAGTAATGTGTGTGCATTTTTCGTCCGGATCACGGCTCCATA
AAATAAATCATATCACAGGGCTCAGGGATTTGTGAGTAGAAGTTCCCGCCACTTAAACGTCACTTGTGAAATCGC
CATCGACGGTCAACTTTTACTCACACTGGTCTTTCGCGGACGGACCGTCTCAAAGTACTGCCTTTCTGCGTTGGA
AAACATCGCCTTTTTCGTCCAAAAGGAGTCCCCAGGTTCGATCCGCATGGCGTTGTGCGTGCGTGCCTTTCTTTT
CAAATGATTACGGCTATTAACTTGGGGGCGTTAAGTTGGAAACACGTAAATTGCAGACTGCGATTAGAGTGACCA
TGAGTAGGAGTTCAAAATCTCCTGACATCATTTTCTTAAAACCTGCTTTGTTTTTTACATTTCTATTTAATATAA
CTCCTATTTGAATAAAAAAACAAAACAAGTTTAGATGTTAAGATATTAACTACATCCTTTGCTCCAAAGGGAGAG
GGGAAGTTATGGAGTTAATTAATTTGCTGTTGGAAATCAATATGGAGTCAGAAATATAATGATTTACTAAACCTT
ATTGAATCGGTAACGATGCGAATTTATATTAAAATAGCTTTTATGAAACATTCAACAAAAATATTATTAATGTTG
GCCCACTTTAGCAACCGGTTAGGTCTACCGGTTGGGCAAGCAAAGATTCACGCCCTGGTTCGAGTCCCAACTAGT
CCTGCAAAATACCGCAGCAAGTTTTAGAGAGACCAAGTGCCATTACCTCTCCCACTTCAGTTATCGGTTATGCGG
CGTTTAAGTCGACAGCTTGCCGTCTCTAGCTCCGGTGCCTATATAAAGCAGCCCGCTTTCCACATTTCATATTCG
TTTTACGTTTGTCAAGCCTCATAGCCGGCAGTTCGAACGTATACGCTCTCTGAGTCAGACCTCGAAATCGTAGCT
CTACACAATTCTGTGAATTTTCCTTGTCGCGTGTGAAACACTTCCAATAAAAACTCCTGCAGGTGAGTACTTTAA
AAAAAAATCTAGTGAAATAATGCTGAAAAGAAATTTGTGTGGGCAAAATTCAATGGGCAAAAACGCGATGCGGCT
TTTTCTCAAAATGGCGGCCGGCCTGCGTTTTTTCCTCAAAAGTGATGACGTCATGCCTGTTTTTTTTTTTTTGTT
CGCAATGAGGAATGGCTCTTAAAATCTACTAGATAAAAAAAATATTCATTATTTCTATGCTGCTGGAACGCTTCA
TTAATCTTAAAAATTCTAAATTCGGTTACCATGATACTTCGACGCATAACTGTAGATTTTGGATAGAATTAAAGA
GAAAATGGCGAGAGAGTAAAATTCCGGCGTCGGCAAAGTAGAGCAAAAAAATCAGTATACCATTTAGCTACCTCT
CTCACTCGCACGCAGTGCCGGCTCAAGTTGGGCGCGGCTCTGCAATTATCGATTTTCTTGGGGTGTGTAACTAAT
*CATCCGTTTTCCCTTCCTCCTCATCCACAGCGTGAA*GGTACCGCGGCCCGGGATCCACCGGTAATCAAC**ATGCC**
ACCCAAGAAGAAGCGAAAAGTAATGATCAGTCTGATTGCGGCGTTAGCGGTAGATCACGTTATCGGCATGGAAAA
CGCCATGCCGTGGAGCCTGCCTGCCGATCTCGCCTGGTTTAAACGCAACACCTTAAATAAACCCGTGATTATGGG
CCGCCATACCTGGGAATCAATCGGTCGTCCGTTGCCAGGACGCAAAAACATTATCCTCAGCAGTCAACCGAGTAC
GGACGATCGCGTAACGTGGGTGAAGTCGGCGGATGAAGCCATCGCGGCGTGTGGTGACGTACCAGAAATCATGGT
GATTGGCGGCGGTCGCGTTTATGAGCAGTTCTTGCCAAAAGCGCAAAAACTGTATCTGACGCATATCGACGCAGA
AGTGGGAGGCGACACCCATTTCCCGGATTACGAGCCGGATGACTGGGGATCGGTATTCAGCGAATTCCACGATGC
TGATGCGCAGAACTCTCACAGCTATTGCTTTGTGATTCTGGGGCGGCGA**ATGGATTACAACAAAAGGAGTAGTGT**
GAGTACGGTGCCGAATGCTGCTCCCATTCGCGTGGGCTTCGTGGGATTGAACGCGGCTAAGGGTTGGGCCATTAA
AACGCATTATCCAGCCATACTGCAGCTGAGCTCCCAGTTCCAAATAACAGCGCTTTATTCCCCCAAGATCGAGAC
GTCCATTGCGACAATTCAGAGGCTGAAGTTGTCCAACGCCACAGCTTTCCCAACACTCGAGAGCTTCGCCTCGAG
CTCCACGATCGATATGATTGTGATCGCAATCCAGGTGGCTTCCCACTACGAGGTGGTAATGCCCCTGCTCGAGTT
TTCGAAGAACAATCCTAATCTGAAGTATTTGTTTGTGGAATGGGCACTGGCCTGCTCGCTCGATCAGGCGGAGTC
GATCTATAAGGCCGCTGCAGAGCGGGGCGTGCAAACAATAATCAGTCTGCAGGGACGAAAGAGCCCGTACATTTT
GCGGGCAAAAGAGCTTATCTCCCAGGGCTACATCGGCGATATAAATAGCATCGAGATTGCAGGTAACGGAGGTTG

GTACGGTTACGAGCGGCCGGTGAAAAGCCCGAAATACATTTACGAGATCGGAAACGGAGTTGATCTGGTGACCAC
CACGTTCGGTCACACGATAGATATATTGCAGTACATGACCAGTTCGTACTTCAGCCGTATCAATGCCATGGTGTT
TAACAACATTCCAGAACAGGAGCTCATTGATGAGAGGGGCAATCGCCTGGGCCAGCGGGTCCCAAAGACTGTCCC
AGATCATCTCTTGTTCCAGGGCACGCTGCTCAACGGAAATGTTCCTGTGTCCTGTTCCTTTAAGGGAGGCAAGCC
TACGAAAAAGTTCACCAAGAATCTGGTCATAGATATCCATGGTACAAAGGGTGATCTGAAGCTGGAGGGTGACGC
TGGATTTGCGGAAATCTCGAATCTCGTTCTGTATTATTCGGGCACCCGCGCCAATGATTTTCCCCTGGCCAATGG
TCAGCAGGCACCCTTGGACCCCGGCTACGACGCAGGCAAGGAGATTATGGAGGTGTATCACCTCCGAAACTACAA
CGCGATCGTTGGTAATATACACCGGCTCTACCAGAGTATCAGCGATTTCCACTTCAACACCAAGAAAATCCCCGA
GCTTCCATCGCAGTTCGTAATGCAAGGCTTCGATTTCGAAGGCTTTCCCACCCTGATGGATGCCTTGATCTTGCA
TCGCCTGATTGAAAGTGTCTACAAGTCGAATATGATGGGCAGTACCCTGAATGTTTCCAACATCTCCCACTACTC
GCTTATGATCAGTCTGATTGCGGCGTTAGCGGTAGATCACGTTATCGGCATGGAAAACGCCATGCCGTGGAGCCT
GCCTGCCGATCTCGCCTGGTTTAAACGCAACACCTTAAATAAACCCGTGATTATGGGCCGCCATACCTGGGAATC
AATCGGTCGTCCGTTGCCAGGACGCAAAAACATTATCCTCAGCAGTCAACCGAGTACGGACGATCGCGTAACGTG
GGTGAAGTCGGCGGATGAAGCCATCGCGGCGTGTGGTGACGTACCAGAAATCATGGTGATTGGCGGCGGTCGCGT
TTATGAGCAGTTCTTGCCAAAAGCGCAAAAACTGTATCTGACGCATATCGACGCAGAAGTGGGAGGCGACACCCA
TTTCCCGGATTACGAGCCGGATGACTGGGGATCGGTATTCAGCGAATTCCACGATGCTGATGCGCAGAACTCTCA
CAGCTATTGCTTTGTGATTCTGGGGCGGCGAGGCGGCCGCGAAGGACGCGGCAGCCTACTGACTTGCGGAGATGT
CGAAGAGAACCCTGGCCCTGGTTCCGATATCTCTAGAGCCACC*GTGTCCAAGGGCGAAGAGGACAACATGGCCAT*
CATCAAGGAGTTCATGCGGTTCAAGGTGCACATGGAAGGCAGCGTGAACGGCCACGAGTTCGAGATCGAGGGCGA
GGGCGAAGGCAGGCCCTACGAGGGCACCCAGACCGCCAAGCTGAAGGTGACCAAGGGCGGACCTCTGCCCTTCGC
CTGGGACATCCTGAGCCCCCAGTTCATGTACGGCAGCAAGGCCTACGTGAAGCACCCCGCCGACATCCCCGACTA
CCTGAAGCTGTCCTTCCCCGAGGGATTCAAATGGGAGCGGGTGATGAACTTCGAGGACGGCGGCGTGGTGACCGT
GACCCAGGACAGCAGCCTGCAGGACGGCGAGTTCATCTACAAGGTGAAACTGCGGGGCACCAACTTCCCCAGCGA
CGGCCCCGTGATGCAGAAAAAGACCATGGGCTGGGAGGCCAGCAGCGAGCGGATGTACCCCGAGGATGGCGCCCT
GAAGGGCGAGATCAAGCAGCGGCTGAAGCTGAAGGATGGCGGCCACTACGACGCCGAGGTGAAAACCACCTACAA
GGCCAAGAAACCCGTGCAGCTGCCTGGCGCCTACAACGTGAACATCAAGCTGGACATCACCAGCCACAACGAGGA
CTACACCATCGTGGAGCAGTACGAGCGGGCCGAGGGCAGACACAGCACCGGCGGCATGGACGAGCTGTATAAATG
AAGCTTAGACATGATAAGATACATTGATGAGTTTGGACAAACCACAACTAGAATGCAGTGAAAAAAATGCTTTAT
TTGTGAAATTTGTGATGCTATTGCTTTATTTGTAACCATTATAAGCTGCAATAAACAAGTTAACAACAACAATTG
CATTCATTTTATGTTTCAGGTTCAGGGGGAGGTGTGGGAGGTTTTTTAAAGCAAGTAAAACCTCTACAAATGTGG
TATGGCTGATTATGATCACATATG**GTACGCGCCCGGGGAGCCCAAGGGCACGCCCTGGCACCCG**

Supplementary Figure 1. The DNA sequences of single and double architecture drug stabilizable GAL80^{ds}. Each architecture has the same *attB40* recombinase site for ΦC31 recombinase mediated transgenesis, constitutively driven by *αTub84B* tubulin promoter, *SV40* nuclear localization signal (NLS), *GAL80* inhibitor of *GAL4* transcriptional activator, *T2A* 2A self-cleaving peptide, *mCherry* fluorescent marker and *SV40* late polyadenylation signal, but one or two copies of *DHFR22-DD*. These single and double architecture *GAL80^{ds}* transgene sequences were verified by Sanger sequencing and subsequently used for generating transgenic flies. **(a)** Single architecture drug stabilizable GAL80^{ds}, DHFR-22 DD fusion to N-terminus of GAL80 (DD-GAL80) sequence. **(b)** Double architecture drug stabilizable GAL80^{ds}, DHFR-22 DD fusion to both N- and C-termini of GAL80 (DD-GAL80-DD) sequence.
